# Factors associated with dental visit and barriers to utilisation of oral health care services in a sample of antenatal mothers in Hospital Universiti Sains Malaysia

**DOI:** 10.1186/1471-2458-10-75

**Published:** 2010-02-18

**Authors:** Norkhafizah Saddki, Azizah Yusoff, Yew L Hwang

**Affiliations:** 1 School of Dental Sciences, Universiti Sains Malaysia, Health Campus, 16150 Kubang Kerian, Kelantan, Malaysia

## Abstract

**Background:**

The aims of this study were to determine factors associated with dental visit and to describe barriers to utilisation of oral health care services among antenatal mothers attending the Obstetric and Gynaecology Specialist clinic in Hospital Universiti Sains Malaysia.

**Methods:**

A structured, self-administered questionnaire was used obtain information on the variables of interest pertaining to the current pregnancy from 124 antenatal mothers.

**Results:**

The majority of the mothers claimed that their oral health status was good (67.0%) or very good (2.4%). On the contrary, most of them admitted of having had at least one oral health problem (59.7%) including cavitated (43.5%) and painful teeth (15.3%), bleeding gum (21.0%), and bad breath (10.5%). However, only 29% of the mothers visited dentist during the current pregnancy. Factors associated with the mothers' dental visit were exposure to oral health education before the pregnancy and awareness of relationship between poor maternal oral health and adverse pregnancy outcomes with odds ratio of 4.06 (95% CI: 1.67-9.78) and 3.57 (95% CI: 1.30-9.77) respectively. Common excuses given by most mothers include perceptions of not having any oral health problems (65.9%), long waiting time at the clinic (71.6%), and no immediate treatment given by the dentist (64.8%).

**Conclusions:**

Utilisation of oral health care services among antenatal mothers was low. Mothers who reported dental visit were more likely to be those who had received oral health education before the current pregnancy and knew of the association between poor maternal oral health and adverse pregnancy outcomes. Dissatisfaction with the services rendered and perceptions of not having any oral health problems were the main barriers.

## Background

Pregnancy is characterized by complex physical and physiological changes that have significant impact on almost every organ system of the body, including the oral cavity. Hormonal, immunologic, dietary, and behavioural changes associated with pregnancy are believed to be the contributing factors. Increased levels of sex hormones, oestrogen and progesterone, are related to increased permeability of oral vasculatures and decreased host immunocompetency. This would then increase the tendency and severity of oral inflammation in reaction to bacterial, physical and chemical irritations [1-3]. Changes in the oral cavity during pregnancy, particularly those involving the periodontium, have been well documented [4,5]. Prevalence of oral mucosal lesions like candidiasis was also reported to be higher in pregnant than non-pregnant women [6].

Periodontal infections during pregnancy do not only affect the mother, but may also bring harm to the foetus if left untreated. Numerous studies had shown that maternal periodontitis increased the likelihood of adverse pregnancy outcomes like premature deliveries, preterm low birth weight and low birth weight infants [7-10]. Some intervention studies also documented that mechanical periodontal therapies such as scaling and root planing during the second trimester of pregnancy may reduce the risk of these adverse outcomes [11-14].

In many countries, specific programs and activities are introduced to improve oral health of women during pregnancy and to educate them about the oral health care of their children. In Malaysia, oral health care is provided by both public and private sectors, and the Ministry of Health is the lead agency in providing care to the public. Oral health program for antenatal mothers has been in place since the early 1970 s [15]. All mothers attending the Maternal and Child Health clinics for antenatal check-ups are referred to the dental clinic for oral health examination and oral health education. Being one of the target groups for oral health care, treatment for the mothers is rendered free of charge in all public health clinics. However, the uptake of oral health care services among the antenatal mothers has been unsatisfactory. The Ministry of Health data from year 1996 to 2002 revealed that only less than a quarter of antenatal mothers attending the Maternal and Child Health clinics had benefited from the oral health care program [15]. In year 2007, the uptake of oral health care services was only 19.7% [16]. Reports of dental care use during pregnancy in other parts of the world also showed dismal figures that ranged from 27% to 61% [17-24].

Several reasons had been cited as barriers to seeking oral health care services among antenatal mothers. These include fear and anxiety of the treatment, low perception for dental problems and treatment, and misconception regarding effects of dental treatment on the developing foetus [17,18,23]. In efforts to improve the deliveries of oral health care services to antenatal mothers, perceived barriers to utilisation of care must first be identified. The specific objectives of this cross sectional study was thus to determine the utilisation of oral health care services and factors associated with dental visit, as well as to describe barriers to utilisation of oral health care services among antenatal mothers attending the Obstetric and Gynaecology Specialist clinic in Hospital Universiti Sains Malaysia.

## Methods

The reference population for this study was antenatal mothers attending public health care services in Kota Bharu, Kelantan, and the source population consisted of pregnant women who received antenatal care from the Obstetric and Gynaecology (O&G) Specialist Clinic in Hospital Universiti Sains Malaysia (HUSM), Kelantan during the months of May and June 2008. In general, HUSM functions mostly as teaching hospital and referral center for cases that require more complex management. However, as a government institution, the basic medical and health care services like antenatal care are accessible to the public at large. The antenatal services are also provided without charge as those available at any other public health care facilities in the country. The ethical approval to conduct this study was obtained from the Research and Ethics Committee (Human), Universiti Sains Malaysia.

The criteria for inclusion in this study were antenatal mothers in the third trimester of gestational period, able to read and write in Malay language and have no display of clear cognitive disturbances. Sample size calculation was done using the formula to estimate a single proportion with requirement for 95% confidence [25]. The prevalence was estimated as 61% based on the results of a study on dental attendance in a sample of pregnant women in Birmingham, United Kingdom [17]. Considering the available resources, a sample size of 113 was selected with a precision of 0.09 (9%). To accommodate for 10% non response rate, a total of 124 antenatal mothers were invited to participate in this study.

In general, antenatal mothers are seen at the clinic on appointment basis but accommodations are made for walk-in patients as well. As such it is not possible to have a sampling frame, and systematic random sampling method was applied for selection of study subjects to get the prior determined sample size. The sampling interval was decided, and every third patient was invited to participate. No possible biases regarding the selection of study subjects were anticipated and the samples were representative of the reference population.

The main outcome of interest in this study was dental visit which was defined as visit to dentist, either in a government clinic or a private practice, for any reason during the current pregnancy. A structured, self-administered questionnaire was used to assist in gathering information on the variables of interest as follows:

a. Socio-demographic and obstetric profile: age, ethnic group, highest education level, household income, employment status, period of gestation, and gravida status.

b. Oral health status during the current pregnancy: perceived oral health status and self-reported oral health problem.

c. Oral health knowledge: had received oral health education prior to current pregnancy, sources of oral health education, knowledge of common oral health problems during pregnancy, and association between poor maternal oral health status and adverse pregnancy outcomes.

d. Access to oral health care services: awareness of free dental services in government clinics, distance to the nearest clinic, perceived accessibility, visit to dentist during current pregnancy, reasons for visit, duration of pregnancy during the first visit, and type of practice visited.

e. Barriers to utilisation of dental services: misperceptions or misconceptions regarding dental visit, dental fears, problems with accessibility, time constraint, and dissatisfaction with the quality of oral health care services at government dental clinics.

The questionnaire was pre-tested prior to the study to ensure its clarity and comprehensiveness. Feedbacks regarding difficulties in understanding and answering the questionnaire were obtained and addressed accordingly. Written informed consent was obtained from all mothers prior to distribution of the questionnaires.

Data entry and analyses of results were done using the Statistical Package for Social Sciences (SPSS) for Windows software (version 12.0; SPSS Inc, Chicago). Data were checked and cleaned. Descriptive statistics such as mean and standard deviation (SD) for continuous variables, and frequency and percentage for categorical variables were determined. The chi-square test was used to compare the profiles of mothers who visited dentist during the current pregnancy with those who did not. The level of significance was set at 0.05.

Factors associated with the mothers' visit to dentist were determined at both univariable and multivariable level using simple logistic regression analysis and multiple logistic regression analysis respectively. In multiple logistic regression analysis, variables for inclusion in the model were selected using forward stepwise logistic regression methods. Following the fit of the preliminary model, the importance of each variable included was verified. The interactions terms were checked using the Likelihood Ratio (LR) test. Multicollinearity problem was identified by the Variance Inflation Factor (VIF) test. The final model was assessed for fitness using the Hosmer-Lemeshow goodness-of-fit test. The classification table for sensitivity and specificity as well as the area under the Receiver Operating Characteristic (ROC) curve were also obtained to evaluate the model fitness. Influential outliers were identified using the Cook's distance. Data point above 1.0 is considered as influential outlier.

## Results

None of the antenatal mothers refused to participate in this study, giving a full response rate of 100%. The socio-demographic and obstetric profiles of the mothers are shown in Table 1. The age of the mothers ranged from 19 to 45 years with a mean age of 31.1 years (SD 5.81). Most of them were of the Malay ethnic group (94.4%). The majority of the mothers have a job (66.1%) and contributed to their household income. At the time of this study, most of mothers (41.1%) were in the early third trimester and 27.4% were expecting their first child.

**Table 1:** Socio-demographic and obstetric profiles of mothers (n = 124)

Variables	Frequency (%)
Age (years)	
≤20	5 (4.0)
21-25	15 (12.1)
26-30	40 (32.3)
31-35	32 (25.8)
36-40	25 (20.2)
>40	7 (5.6)
	
Ethnic group	
Malay	117 (94.4)
Others	7 (5.6)
	
Highest educational level	
Secondary	57 (46.0)
Post-secondary/Diploma	36 (29.0)
Tertiary	31 (25.0)
	
Household income (Malaysian Ringgit)	
<1000	24 (19.4)
1000-3000	62 (50.0)
3001-5000	25 (20.2)
>5000	13 (10.5)
	
Employment status	
No	42 (33.9)
Yes	82 (66.1)
	
Period of gestation (weeks)	
28-32	51 (41.1)
33-37	46 (37.1)
>37	27 (21.8)
	
Gravida status	
Primigravida	34 (27.4)
Multigravida	90 (72.6)

Perceived oral health status of the mothers is shown in Table 2. The majority of the mothers claimed that their oral health status was good (67.0%) or very good (2.4%). On the contrary, most of them also admitted of having had at least one oral health problem (59.7%) during the current pregnancy. Common oral health problems among the mothers include cavitated (43.5%) and painful teeth (15.3%), bleeding gum (21.0%), and bad breath (10.5%). Other oral health problems were also reported as listed.

**Table 2:** Perceived oral health status of mothers (n = 124)

Variables	Frequency (%)
Oral health status	
Very poor	0 (0.0)
Poor	0 (0.0)
Fair	38 (30.6)
Good	83 (67.0)
Very good	3 (2.4)
	
Self-reported oral health problem	
None	50 (40.3)
One problem	39 (31.5)
Two or more problems	35 (28.2)
	
Type of oral health problems (n = 74)	
Toothache	19 (15.3)
Cavitated tooth	54 (43.5)
Gum pain	8 (6.5)
Gum swelling	11 (8.9)
Gum bleeding	26 (21.0)
Gum abscess	1 (0.8)
Loose tooth	7 (5.6)
Bad breath	13 (10.5)

Some mothers (28.2%) had received oral health education prior to their current pregnancy, and mostly (60.0%) were given by their dentists. Other sources of oral health knowledge include magazine (48.6%), television (42.9%), newspaper (40.0%), pamphlet (34.3%), radio (22.9%), and internet (20.0%). Interestingly, 14.3% had received oral health education from their medical doctors. Most mothers were able to identify dental caries (63.7%) and periodontal disease (50.8%) as the common oral health problems during pregnancy. In addition, more than half of the mothers were aware of the association between poor maternal oral health and adverse pregnancy outcomes (61%).

Most of the mothers knew that oral health services for antenatal mothers are available at no charge in government clinics (57.3%). Estimation of distance between their homes to the nearest dental clinic showed a positively skewed distribution. The central tendency is thus given in median and the dispersion is given in inter-quartile range (IQR). The median distance was 5.0 km (IQR 5.90) with the minimum and maximum distance of 0.5 km and 29.0 km respectively. Most of the mothers claimed that it would be either easy (72.6%) or very easy (21.0%) for them to visit the nearest dental clinic while few others considered the visit to be difficult (6.4%). Of 124 mothers, only 36 (29%) mothers visited dentist during the current pregnancy.

The reasons for visit vary, and some mothers gave more than one reason for their visit (36.1%). The most frequent reasons for dental visit were toothache (58.3%) and gum problems (38.9%). Other problems that prompted visit include bad breath (5.6%) and mobile tooth (5.6%). Some were referred by their doctors or nurses (13.9%), and commendably, there were mothers who visited dentist for routine check-up (30.6%). Most of the mothers visited dentist during their first trimester (50.0%). Others went during their second and third trimester, 36.1% and 13.9% respectively. The majority of the mothers preferred dentists in private practices (58.3%) compared to the government clinics.

Table 3 shows the distribution of the mothers' profile in the group who visited dentist as compared with those who did not. Owing to the small number of responses in some categories, few of the variables were regrouped to fulfil the chi-square test assumptions that no expected frequency should be less than 1 and no more than 20% of the expected frequencies should be less than 5. The results show that the prevalence of mothers who had received oral health education prior to the current pregnancy and who were aware of the association between poor maternal oral health and adverse pregnancy outcomes were significantly different between the two groups. The influence of socio-demographic background as well as obstetric profile on dental visit was not apparent. Similarly, perceived oral health status, self-reported oral health problems, awareness of availability of free dental services in government clinics, distance from home to clinic, and perceived accessibility to dental clinic were not significantly different between the two groups.

**Table 3:** Comparisons of the profiles of mothers who visited dentist during the current pregnancy (n = 36) with those who did not (n = 88)

Variables	Dental visit		**χ**^**2**^**statistics (df)**	p value
			
	Yes (%) n = 36	No (%) n = 88		
Age (years)				
≤35	25 (69.4)	67 (76.1)	0.598 (1)	0.440
> 35	11 (30.6)	21 (23.9)		
				
Ethnic group				
Malay	35 (97.2)	82 (93.2)	-	0.672^a^
Others	1 (2.8)	6 (6.8)		
				
Highest educational level				
Secondary	19 (52.8)	38 (43.2)	3.810 (2)	0.149
Post-secondary/Diploma	6 (16.7)	30 (34.1)		
Tertiary	11 (30.6)	20 (22.7)		
				
Household income (Malaysian Ringgit)				
<3000	25 (69.4)	61 (69.3)	0.331 (2)	0.848
3001-5000	8 (22.2)	17 (19.3)		
>5000	3 (8.3)	10 (11.4)		
				
Employment status				
No	12 (33.3)	30 (34.1)	0.007 (1)	0.936
Yes	24 (66.7)	58 (65.9)		
				
Period of gestation (weeks)				
28-32	15 (41.7)	36 (40.9)	0.440 (2)	0.803
33-37	12 (33.3)	34 (38.6)		
>37	9 (25.0)	18 (20.5)		
				
Gravida status				
Primigravida	10 (27.8)	24 (27.3)	0.003 (1)	0.954
Multigravida	26 (72.2)	64 (72.7)		
				
Oral health status				
Fair	13 (36.1)	25 (28.4)	0.713 (1)	0.398
Very good/good	23 (63.9)	63 (71.6)		
				
Self-reported oral health problem				
None	11 (30.6)	39 (44.3)	2.375 (2)	0.305
One problem	12 (33.3)	27 (30.7)		
Two or more problems	13 (36.1)	22 (25.0)		
				
Oral health education received before the current pregnancy				
No	17 (47.2)	72 (81.8)	15.094 (1)	<0.001
Yes	19 (52.8)	16 (18.2)		
				
Aware of relationship between maternal oral health and pregnancy outcomes				
No	6 (16.7)	42 (47.7)	10.389 (1)	0.001
Yes	30 (83.3)	46 (52.3)		
				
Aware of free dental services in government clinics				
No	11 (30.6)	42 (47.7)	3.078 (1)	0.079
Yes	25 (69.4)	46 (52.3)		
				
Distance to nearest dental clinic (km)				
≤5	20 (55.6)	60 (68.2)	1.779 (1)	0.182
>5	16 (44.4)	28 (31.8)		
				
Accessibility to the nearest dental clinic				
Difficult	2 (5.6)	6 (6.8)	0.102 (2)	0.950
Easy	26 (72.2)	64 (72.7)		
Very easy	8 (22.2)	18 (20.5)		

^a ^Fisher's Exact test

Table 4 shows the results of simple logistic regression analysis of factors associated with the mothers' visit to dentist. No significant association was found between dental visit and the mothers' age, ethnic group, education level, household income, employment status, period of gestation, gravida status, perceived oral health status, self-reported oral health problems, awareness of free dental services in government clinics, distance from home to clinic, and perceived accessibility to dental services. However, significant association was found between the mothers' visit to dental clinic and oral health education received before the current pregnancy and awareness of the association between poor maternal oral health and adverse pregnancy outcomes with odds ratio (OR) of 5.03 (95% CI: 2.15-11.76) and 4.57 (95% CI: 1.73-12.06) respectively.

**Table 4:** Factors associated with dental visit among mothers by simple logistic regression analysis

Variables	Crude OR	95% CI	**LR χ**^**2**^**(df)**^a^	**p value**^a^
Age (years)				
≤35	1.00	--	0.593 (1)	0.444
>35	1.40	0.59-3.32		
				
Ethnic group				
Malay	1.00	--	0.891 (1)	0.345
Others	0.39	0.05-3.37		
				
Highest educational level				
Secondary	1.00	--	4.078 (2)	0.130
Post-secondary/Diploma	0.08	0.14-1.12	3.010 (1)^b^	0.083^b^
Tertiary	0.84	0.44-2.76	0.041 (1)^b^	0.839^b^
				
Household income (Malaysian Ringgit)				
<3000	1.00	--	0.339 (2)	0.844
3001-5000	1.15	0.44-3.00	0.080 (1)^b^	0.778^b^
>5000	0.73	0.19-2.89	0.199 (1)^b^	0.656^b^
				
Employment status				
No	1.00	--	0.007 (1)	0.936
Yes	1.03	0.46-2.35		
				
Period of gestation (weeks)				
28-32	1.00	--	0.437 (2)	0.804
33-37	0.85	0.35-2.07	0.133 (1)^b^	0.715^b^
>37	1.20	0.44-3.27	0.127 (1)^b^	0.721^b^
				
Gravida status				
Primigravida	1.00	--	0.003 (1)	0.954
Multigravida	0.98	0.41-2.32		
				
Oral health status				
Fair	1.00	--	0.701 (1)	0.400
Very good/good	0.70	0.31-1.60		
				
Self-reported oral health problem				
None	1.00	--	2.390 (2)	0.303
One problem	1.58	0.61-4.09	0.873 (1)^b^	0.350^b^
Two or more problems	2.10	0.80-5.46	2.289 (1)^b^	0.130^b^
				
Oral health education received before the current pregnancy				
No	1.00	--	14.334 (1)	<0.001
Yes	5.03	2.15-11.76		
				
Aware of relationship between maternal oral health and pregnancy outcomes				
No	1.00	--	11.271 (1)	0.002
Yes	4.57	1.73-12.05		
				
Aware of free dental services in government clinics				
No	1.00	--	3.150 (1)	0.082
Yes	2.08	0.91-4.73		
				
Distance to nearest dental clinic (km)				
≤5	1.00	--	1.749 (1)	0.184
>5	1.71	0.77-3.80		
				
Accessibility to the nearest dental clinic				
Difficult	1.00	--	0.104 (2)	0.950
Easy	1.22	0.23-6.44	0.054 (1)^b^	0.816^b^
Very easy	1.33	0.22-8.10	0.098 (1)^b^	0.755^b^

^a ^Likelihood Ratio (LR) test

^b ^Wald test

At multivariable level, oral health education received and awareness of relationship between maternal oral health and pregnancy outcomes remain as factors significantly associated with the mothers' dental visit with OR of 4.06 (95% CI: 1.67-9.78) and 3.57 (95% CI: 1.30-9.77) respectively. Table 5 shows results of multiple logistic regression analysis. Possible two-way interactions between factors were not significant, and there was no multicollinearity problem. The preliminary final model was checked for fitness. The results of Hosmer-Lemeshow goodness-of-fit test was not significant (*p *= 0.994, df = 2) and the area under the ROC curve was 0.737, suggesting that the model was fit. The sensitivity and specificity of this model was 47.2% and 87.5% respectively. The overall correct classification result indicated that 75.8% of the mothers are predicted correctly whether they visit the dentist or not. Contribution of each outlier was checked, and none was found to be influential.

**Table 5:** Factors associated with dental visit among mothers by multiple logistic regression analysis

Variables	Adjusted OR	95% CI	**LR χ**^**2**^**(df)**^a^	**p value**^a^
Oral health education received before the current pregnancy				
No	1.00	--	9.940 (1)	0.002
Yes	4.06	1.69-9.78		
				
Aware of relationship between maternal oral health and pregnancy outcomes				
No	1.00	--	6.877 (1)	0.009
Yes	3.57	1.30-9.77		

^a ^Likelihood Ratio (LR) test

Barriers cited by the mothers who did not go for dental visit were grouped into five key areas and are listed in Table 6. Various reasons were given by the mothers for not going to the dentists. The combinations of reasons were extremely extensive, and the majority encompassed two or more key areas (84.1%). However, the common reasons given by most of the mothers ranged from perceptions of not having any oral health problems (65.9%), long waiting time at the clinic (71.6%), and no immediate treatment given by the dentist (64.8%). Some of the mothers responded that they were busy either at work (38.6%) or with the household chores (30.7%), as well as reasons concerning their difficulties to access the dental clinic as well as their dental fears.

**Table 6:** Barriers to utilisation of oral health care services among mothers who did not visit dentist (n = 88)

Variables	Frequency (%)
Misperceptions/misconceptions	
Not having any dental problems	58 (65.9)
Oral health is not/less important	9 (10.2)
Negative effect of treatment on fetus	14 (15.9)
	
Dental fears	
Fear to dentist	1 (1.1)
Fear to dental instrument	9 (10.2)
Fear to dental treatment	10 (11.4)
Fear to dental pain	25 (28.4)
Bad dental experience	8 (9.1)
	
Accessibility to dental clinic	
Long distance	12 (13.6)
Long travelling time	4 (4.5)
No self transport	6 (6.8)
Difficult to get a public transport	5 (5.7)
Expensive fee for public transport	2 (2.3)
	
Time constraints	
Busy at work	34 (38.6)
Unable to get permission for leave from employer	13 (14.8)
Busy with household chores	27 (30.7)
	
Dissatisfaction with the quality of services	
Late appointment	33 (37.5)
Long waiting time	63 (71.6)
No immediate treatment given	57 (64.8)
Poor service at the registration counter	9 (10.2)
Poor attitude of the staff	7 (8.0)
Poor attitude of the dentist	4 (4.5)
Poor condition of the treatment room	4 (4.5)
Poor confidence with the services rendered	8 (9.1)

## Discussion

Antenatal care has long been endorsed as the means to improve pregnancy outcomes by promoting preventive health care and healthy behaviours. Given the wide array of problems associated with pregnancy, good oral health care is essential for antenatal mothers. However, the important contribution of oral health to antenatal care is still not widely appreciated. An integrated and comprehensive approach to antenatal care requires oral health to be a compulsory component in the complex of interventions that a pregnant woman receives from any organized health care services. It means that dental examination should form the basic activities of antenatal care along with blood pressure monitoring, weight gain assessment, and obstetric examination. It is quite distressing, however, that the World Health Organization has not included oral health care as one of the basic component in the new antenatal care model [26].

Studies had shown that most women do not attend dental services during pregnancy. In the United States, analysis of the Pregnancy Risk Assessment Monitoring System (PRAMS) data in 1998 from the states of Arkansas, Illinois, Louisiana, and New Mexico revealed that only between 23% to 35% of pregnant women reported dental care use during pregnancy [20]. In another analysis of PRAMS data from Washington, dental visits during pregnancy were reported by 42% of respondents [24]. Other studies in the United States also showed that less than half of women visited dentist during their pregnancy [21,23].

In the United Kingdom, oral health care forms an integral part of antenatal care. Dental services are exempted from the National Health Services fee to all pregnant mothers up until 12 months post-partum. However, a study done among a sample of immigrant women in North London showed an attendance rate of only 32% [19]. In Northern Greece, only 27.3% of women reported visit to the dentist during their pregnancy [18], and in Kuwait, just a bit more than half of antenatal mothers (52%) utilized dental services [22]. In this study, the utilisation of oral health care services among the antenatal mothers was 29%.

It was noted that this prevalence was higher than that ever reported by the Ministry of Health Malaysia [15]. The most possible explanation for this is that the uptake of services reported by the Ministry of Health was obtained by dividing the number of new antenatal attendances to government dental facilities by the total number of new antenatal attendances to the Maternal and Child Health clinics in the particular year. Data obtained are used by the Ministry to monitor and evaluate the oral health care program as well as to facilitate in planning for resources. Thus, only visits to the government facilities were counted, whereas in this study, the definition of dental visit comprises of visit to both the government and the private clinics. Given the fact that more mothers preferred private dentists in this study, it is therefore appropriate that a higher prevalence was obtained. However, considering that the attendance rate for antenatal care in Malaysia from year 2000 to 2007 was only 79% [27], the problems of poor utilisation of dental services may be bigger than that revealed in this study.

Poor socio-economic conditions as indicated by low education level, unemployment and small household income, are important factors found to be associated with lesser likelihood of not going for a dental visit during pregnancy [20,21,24]. However, the influence of these factors on mothers' dental visit was not apparent in this study. Similar findings were reported by Dinas *et al*. [18]. Instead, the situation seems to be in reverse in this study where the prevalence of dental visit was lower in mothers with tertiary education, who were working, and had higher household income. The most logical explanation for this is that higher education may lead to job with better salaries that keep the mothers very busy. Although the differences were not significant, further investigation in this matter may be warranted.

The knowledge that dental treatment is offered free of charge at government dental clinics was not associated with the mothers' dental visit. This finding indicates that the availability of free dental services is not fully appreciated by the mothers such that the services were not utilised to the maximum benefit. This is in contrast to previous report by Dinas *et al*. that mothers who visited dentist during their pregnancy were significantly more likely to know that dental treatment is free of charge [18]. The results of this study also showed that perceived oral health status and self-reported oral health problems did not make a significant difference as to whether or not the mothers visit a dentist. A study by in Kuwait also found that perceived oral health problems was not a factor associated with dental visit among antenatal mothers [22]. Similar results were obtained by other authors [20,23]. These results reflect the mothers' poor attitude towards oral health care that needs to be addressed.

The results of this study revealed that mothers who reported dental visit were more likely to be those who had received oral health education before the current pregnancy and knew of the association between poor maternal oral health and adverse pregnancy outcomes. This is consistent with a study by Al-Habashneh *et al*. that mothers who have heard about the possible connection between oral health and pregnancy were significantly more likely to report dental visit during pregnancy [21]. These findings further established the important roles of oral health education to impart knowledge and increase awareness that would in turn improve the mothers' dental care-seeking behaviour. Hence, the provision of oral health education to all antenatal mothers should be made mandatory in effort to improve uptake of services. Besides, oral health education could be used as a behavioural technique to alleviate dental fear among the mothers by making them more at ease and familiar with the dentist and the forthcoming treatment procedures. All misperceptions and erroneous conception about the safety of dental treatment that may contribute to the low rate of service utilisation can be corrected.

Patient satisfaction with the quality of services is an essential component of health care. It affects patients' use of services, compliance to care, and is also associated with health status and outcomes [28]. Waiting time is an important quality indicator in measuring the outcome of any medical service. Extended waiting time at the dental clinic and substantial delay in receiving the needed care had resulted in patient dissatisfaction with the care provided [29,30]. For most mothers in this study, time was an important essence as reflected in their responses that they were busy either at work or running the household chores. Consequently, 'late appointment' and 'long waiting time' were important barriers that prevent access to oral health care services among them.

Poor dentist to population ratio may contribute to some of the disappointments with the services rendered. The total number of dentists in Malaysia in year 2008 stood at 3,640 that gave an overall dentist to population ratio at 1 to 7,618 [31]. Although there were more dentists in the public sector (52.8%) than in the private practice, a considerable number of them were actually at administrative posts that may reduce the proportion further. Results of this study showed that most mothers visited private dentists for their oral health care during pregnancy. This is possibly because most participants were working mothers and that they may have additional disposable income. Besides, as time is an important limitation for most of them, visiting a private practitioner may be a more convenient option since private clinics are mostly accessible after hours and during weekends. Waiting time at private clinics is also relatively short and treatment can be started immediately [32]. It is therefore timely for the private dentists to take more active role in oral health promotion and services to antenatal mothers. Collaborative efforts between the Ministry of Health and the Malaysian Private Dental Practitioners' Association are recommended so as to ensure delivery of a more accessible oral health care program for antenatal mothers in this country.

Most oral diseases are silent in nature such that people tend to delay treatment. The majority of mothers in this study who did not attend dental clinic claimed that they had no dental problem. In fact, most mothers perceived their oral health status to be good or very good. On the other hand, many of them also reported having problems with their teeth and gum. This implies that the mothers did not perceive their oral health care as an urgent need and would rather delay visit until after delivery. These results concurred with other studies [17,22,23]. Another mistaken belief among mothers in this study was that dental treatment during pregnancy is harmful to the foetus. This misconception was reported as the most important factor limiting access to dental care among antenatal mothers in Northern Greece [18]. Dental fear, particularly to dental pain was also reported by some mothers, and it is well documented that dental fear and anxiety have significant impact on dental care use behaviours [33-35].

The medical doctors and nurses are the front liners in antenatal care. Their responsibilities in oral health care provision are mainly to recommend dental referral to all antenatal mothers and to emphasis the importance of good oral health care. Hence, it is imperative that they too are aware of the current evidence linking maternal oral health and pregnancy outcomes. Conversely, studies have shown that medical practitioners do not regard oral health care as an essential part of antenatal care, and that most of them do not routinely advise their antenatal patients to seek dental care [36,37]. This is apparent from the results of this study that only five mothers (13.9%) were referred by their doctors or nurses for dental visit. As such, continuing education to the medical providers on the current issue is deemed necessary. In addition, there should be mechanisms that can effectively facilitate communication and encourage cross-referral between dental and medical health care providers. Immediate action to revise the existing referral system in this country that has been in place for almost four decades is very much needed.

## Conclusions

This study provides an interesting insight into barriers to utilisation of oral health care services among antenatal mothers. On the other hand, information obtained through self-administered questionnaire has to be interpreted with caution due to bias created through favourable responses. Considering that this study was conducted in a health care setting, the mothers may also have felt compelled to indicate that they attended dental services. In addition, dental examinations were not performed on the mothers, thus it is not possible to determine the actual dental treatment needs. However, within these limitations, this study concludes that oral health care utilisation among antenatal mothers was low. Mothers who reported dental visit were more likely to be those who had received oral health education before the current pregnancy and knew of the association between poor maternal oral health and adverse pregnancy outcomes. Dissatisfaction with the services provided and perceptions of not having any problem were the main barriers. In essence, oral health care seeking behaviour among the antenatal mothers needs to be reformed and the importance of oral health care must be appreciated and translated into actions.

## Competing interests

The authors declare that they have no competing interests.

## Authors' contributions

NKS contributed to the design of the study, data analyses and interpretation. AY contributed to the design of the study and data interpretation. HYL contributed to the data acquisition, data analyses and interpretation. All authors helped to draft the manuscript. All authors read and approved the final manuscript.

## Pre-publication history

The pre-publication history for this paper can be accessed here:

http://www.biomedcentral.com/1471-2458/10/75/prepub
